# Identifying the geographic leading edge of Lyme disease in the United States with internet searches: A spatiotemporal analysis of Google Health Trends data

**DOI:** 10.1371/journal.pone.0312277

**Published:** 2024-11-13

**Authors:** Cara Wychgram, John N. Aucott, Alison W. Rebman, Frank C. Curriero

**Affiliations:** 1 Spatial Science for Public Health Center, Department of Epidemiology, Johns Hopkins Bloomberg School of Public Health, Baltimore, Maryland, United States of America; 2 Lyme Disease Research Center, Division of Rheumatology, Department of Medicine, Johns Hopkins University School of Medicine, Baltimore, Maryland, United States of America; Universita degli Studi di Parma, ITALY

## Abstract

**Background:**

The geographic footprint of Lyme disease is expanding in the United States, which calls for novel methods to identify emerging endemic areas. The ubiquity of internet use coupled with the dominance of Google’s search engine makes Google user search data a compelling data source for epidemiological research.

**Objective:**

We evaluated the potential of Google Health Trends to track spatiotemporal patterns in Lyme disease and identify the leading edge of disease risk in the United States.

**Materials and methods:**

We analyzed internet search rates for Lyme disease-related queries at the designated market area (DMA) level (n = 206) for the 2011–2019 and 2020–2021 (COVID-19 pandemic) periods. We used maps and other exploratory methods to characterize changes in search behavior. To assess statistical correlation between searches and Lyme disease cases reported to Centers for Disease Control and Prevention (CDC) between 2011 and 2019, we performed a longitudinal ecological analysis with modified Poisson generalized estimating equation regression models.

**Results:**

Mapping DMA-level changes in “Lyme disease” search rates revealed an expanding area of higher rates occurring along the edges of the northeastern focus of Lyme disease. Bivariate maps comparing search rates and CDC-reported incidence rates also showed a stronger than expected signal from Google Health Trends in some high-risk adjacent states such as Michigan, North Carolina, and Ohio, which may be further indication of a geographic leading edge of Lyme disease that is not fully apparent from routine surveillance. Searches for “Lyme disease” were a significant predictor of CDC-reported disease incidence. Each 100-unit increase in the search rate was significantly associated with a 10% increase in incidence rates (RR = 1.10, 95% CI: 1.07, 1.12) after adjusting for environmental covariates of Lyme disease identified in the literature.

**Conclusion:**

Google Health Trends data may help track the expansion of Lyme disease and inform the public and health care providers about emerging risks in their areas.

## Introduction

Lyme disease is a bacterial infection whose causative agent is transmitted by the bite of infected *Ixodes* species ticks, which are found widely in northern temperate regions of the world. In the United States, the responsible tick vectors are *Ixodes scapularis* in the east and *Ixodes pacificus* in the west. Most cases are reported in the Northeast, Mid-Atlantic, and Upper Midwest regions and, to a lesser extent, along the Pacific coast [1, 2]. Since Lyme disease was first identified in the late 1970s, its incidence has increased steadily, and it is now the most reported vector-borne disease in the country. Due in part to climate and land use changes, ticks that may carry Lyme disease-causing bacteria are expanding their geographic reach [3], sparking concern about their future impact on human health. In the United States, surveillance of Lyme disease in humans is based on case definitions published by Centers for Disease Control and Prevention (CDC). Cases are reported passively, relying on health care providers or laboratories to initiate reports to health departments, as opposed to being actively sought out. Passive surveillance has been known to suffer from significant underreporting [4]. It is not designed to capture every case and may have low sensitivity for detecting newly affected geographic areas. Moreover, publication of case data is delayed by one or more years, making it difficult to track trends and emerging endemic areas in real time. The impact of the COVID-19 pandemic on Lyme disease infections and case reporting has also been unclear [5].

In recent years, there has been growing interest in data sources that may complement traditional disease surveillance. Data from insurance claims and electronic health records are now commonly used in Lyme disease research [2, 6–10]. Researchers are also turning to search engine and social media data, which are now readily available through application programming interfaces (APIs). These and other internet-based data sources form the toolkit of the emerging fields of infodemiology and infoveillance [11]. Although Twitter data research has grown more recently [12–14], the literature linking internet-based data to Lyme disease has largely focused on Google Trends, a publicly available tool that allows users to look up the relative popularity of a search query. Given that Google commands more than 90% of the search engine market [15], this tool can provide valuable insights into what people are searching for online. Seifter et al. [16] used Google Trends to show that the search query “Lyme disease” was most popular in spring and summer months, when reported cases are known to peak, and in cities and states where the disease is endemic in the United States. Couper et al. [17] found that searches for “ticks” were a statistically significant predictor of Lyme disease incidence between 2004 and 2017, although Google Trends was not the focus of their research. Others have separately explored spatial and temporal trends in Lyme disease-related search queries [18–21]. To our knowledge, no one has investigated spatial trends over time. A spatiotemporal analysis of Google Trends data may provide insights into both where and the degree to which public interest or concern in Lyme disease is changing over time. We hypothesized that the identification of these areas may help detect the “leading edge” [22] of Lyme disease in the United States, or the expanding geographic boundary that separates existing high-risk areas and areas where risk is currently increasing.

In the current study, we utilized data from the Google Health Trends API [23], which has advantages over Google Trends for comparing geographic locations over time, to 1) explore designated market area (DMA)-level trends in searches for “Lyme disease” and “tick bite” for the 2011–2019 and 2020–2021 (COVID-19 pandemic) periods and 2) assess the predictive power of searches on CDC-reported Lyme disease incidence rates between 2011 and 2019.

## Materials and methods

### Materials

#### Lyme disease-related search query data

Although public Google Trends data are commonly used in infodemiology research, they are not appropriate for spatiotemporal analyses because of the way that Google normalizes search volumes and rescales them to a 0–100 index [24]. This indexing makes the data too coarse to allow for meaningful analysis of search behavior at the level needed to assess changes across space and time. Google Health Trends is an alternative resource that is available to researchers through a private API. The API data are quantitatively different from public Google Trends data. They are still normalized but they are not scaled [25], making comparisons across geographic locations and time periods possible. The API returns a query fraction, or search rate, which represents the probability of a search session containing a case-insensitive search query (e.g., “Lyme disease”/”lyme disease”) in a specified location and time period. For readability, the query fraction is presented as the number of searches per ten million searches. The absence of 0–100 scaling also makes repeated sampling more meaningful. Google Health Trends data are based on a random sample of all Google searches, which refreshes daily, so multiple samples may be averaged to obtain a more reliable estimate of a search query’s popularity [24].

We used the R package *gtrendR* [26] to extract annual query fractions for the search queries “Lyme disease” and “tick bite” from the Google Health Trends API. We considered including other Lyme disease-related queries in our study but did not do so for the following reasons. First, most symptom-related queries (e.g., “fever” or “headache”) are not specific to Lyme disease. Second, less commonly searched queries (e.g., “bull’s-eye rash”) tend to return mostly missing values (i.e., no or low search volume) at the DMA level. Finally, the Google Health Trends API’s daily quota limit only allowed us to sample one query’s worth of data per day, so adding more queries would have significantly extended our data collection.

The smallest spatial unit for which Google makes search query data available is the DMA, which is a grouping of counties. There are 206 DMAs in the contiguous United States; they are typically defined by their largest metropolitan area and may include suburbs and other outlying areas. Google Health Trends data date back to 2004, although the literature warns that data quality in earlier years may be worse because internet use was not as widespread and Google faced more competition from other search engines [24, 27–29]. We first dated our API requests back to 2004 to assess early data quality. We determined that both missing and outlier values dropped substantially after 2010; in fact, anomalously large query fractions were no longer present beginning in 2011. Although we could find no other studies that addressed temporal quality of DMA-level Google Health Trends data, our finding was consistent with the literature’s suggestion that pre-2007 and even pre-2014 Google Trends data may be less reliable prior to the introduction of the iPhone and the eclipse of desktop browsing by mobile browsing, respectively [24, 28]. Therefore, we removed pre-2011 observations from the data and restricted our data set to 2011–2021.

We used the same API request parameters to extract multiple samples on different days because the random sample of searches on which Google Trends query fractions are based changes daily. Stephens-Davidowitz & Varian [24] note that researchers may average different samples if very precise results are needed, but that Google’s sample is large enough that different samples should yield similar results. The variability that we observed among our early samples seemed to warrant repeated sampling. We noted a sample size of 30 from a few Google Trends studies that mentioned resampling [30–33], perhaps based on the minimum sample size rule of thumb in statistics, and agreed that 30 was a reasonable number to balance data collection time and confidence in the accuracy of the data.

Each daily sample contained the annual query fractions over all available years (2004–2021) for all 206 DMAs. Before averaging the 30 samples for each DMA-year combination, we inspected the data and noticed that the API sometimes returned missing values (NAs), which are returned when the absolute number of searches in a particular location and time period is below an unreported privacy threshold [24]. Although Google refers to these missing values as zeros, the true query fraction is likely greater than zero. Missing values are more likely to be returned in less populated locations and earlier time periods [24]. We observed this in our samples for earlier years and noted that, in DMAs with many missing values across the 30 samples, the non-missing query fractions were highly variable and, in some cases, anomalously large. In addition to calculating the average of non-missing samples, we tallied the missing samples so that we could assess data quality by year. Surprisingly, some DMAs returned all missing values between 2004 and 2021; the result of absolute searches never meeting the privacy threshold, this meant that we could not calculate any averages for four DMAs in the “Lyme disease” data and 30 DMAs in the “tick bite” data. As expected, these DMAs tended to be less populated and located in low Lyme disease incidence states. The four DMAs with missing “Lyme disease” data were in Texas, Nebraska, and Montana and had no or very low Lyme disease incidence. The 30 DMAs with missing “tick bite” data were located across the country, with the highest number located in the South; Lyme disease incidence in these areas was typically below five cases per 100,000 people. It is likely that a lower frequency of “tick bite” searches compared to “Lyme disease” searches, combined with smaller populations, contributed to the higher number of DMAs with missing “tick bite” data.

Although 30 samples allowed us to explore and address data issues (i.e., outliers and missingness) in an unfamiliar data source; ultimately, when comparing query fraction variability across five samples to variability across 10, 15, and up to 30 samples, more samples were not critical. To test for differences in variability, we randomly selected five, 10, and 15 of the 30 possible samples and calculated the coefficient of variation (CV) of the averaged “Lyme disease” query fractions for each group, including a group with all 30 samples. We then performed a one-way ANOVA test with CV as the dependent variable and number of samples group as the independent variable. We replicated the random sample selection and ANOVA test 1,000 times and averaged the *P* value of all tests. We found no significant difference (*P* = 0.229) in the average CV among groups, which was around 6%. S1 Fig shows the distribution of CV across the four groups for one of the replications. Google’s changing random sample of searches may introduce concerns about sampling error, as Google does not disclose specifics about its sampling design. However, in our case, we agree with Stephens-Davidowitz & Varian [24] that Google’s sample is likely large enough that a single to a few samples give a reliable estimate of “Lyme disease” search rates. Particularly after removing pre-2011 observations from our data, query fractions were not identical, but similar across samples, with some DMAs and years showing more variation than others. We still view repeated sampling as a responsible use of Google Health Trends data given its relative novelty; however, the value gained from repeated sampling likely depends on the researcher’s particular search query, time and location parameters, and methods.

Our use of the Google Health Trends API complied with Google’s terms of service and responsible use guidelines, which included non-commercial use and sharing of raw data in summary form only.

#### Reported Lyme disease incidence rate data

We obtained county-level, CDC-reported Lyme disease cases between 2011 and 2019 from the Johns Hopkins Lyme and Tickborne Diseases Dashboard and aggregated them to the DMA level [34, 35]. Data for 2020–2021 were not yet available at the time of analysis. Nearly all counties were located within a single DMA, but we identified seven counties that were each split between two DMAs. For these counties, we calculated the amount of overlap and for each year assigned a proportion of cases to either DMA (see S1 Table). This was a crude fix, but we did not have enough information (e.g., finer-scale Lyme disease data) to produce more accurate estimates. Only one of the counties (Oneida County, New York) was in the eastern half of the country where Lyme disease is most common; the other six counties were in Arizona, California, and New Mexico and had very few reported cases. We also aggregated county-level population estimates from the Census Bureau [36] to the DMA level to calculate the incidence rate of reported Lyme disease cases per 100,000 people.

#### Environmental data

To determine if a modeled relationship between searches and reported incidence rates remained significant after adjusting for other covariates, we included a selection of environmental factors that have been associated with Lyme disease in the literature [17, 37–41]. These landscape and climate factors may act as limiting factors for ticks and human-tick interactions. For landscape covariates, we used elevation, forest cover, and open space vegetation cover (i.e., lawn grasses and other vegetation in developed settings). Definitions of non-forest vegetation vary across the literature and may depend on study area; we used open space vegetation as our study area was the contiguous United States and other vegetation classes (e.g., herbaceous) have very low coverage in the eastern United States, where Lyme disease is most common, relative to the rest of the country. We also included Normalized Difference Vegetation Index (NDVI) as an annual measure of vegetation density. For climate covariates, we used annual and seasonal temperature and precipitation. The appropriateness of environmental predictors at the DMA level was also a consideration in our selection. For example, a measure of overall forest cover was included, but more detailed forest metrics, such as patch size and edge density, did not seem meaningful when summarized over such a large spatial unit.

We obtained a digital elevation model with 30-m resolution from the United States Geological Survey (USGS), a 2016 land cover classification raster with 30-m resolution from the National Land Cover Database, annual maximum NDVI rasters with 250-m resolution from USGS, and gridded monthly total precipitation and mean temperature data with 4-km resolution from the PRISM Climate Group at Oregon State University [42–44]. We used ArcGIS Pro version 3.1 [45] to calculate DMA-level characteristics including average elevation, percent land cover by class (2016), average maximum NDVI (2011–2019), and annual and seasonal precipitation and average temperature (2011–2019). Seasonal calculations were based on meteorological seasons (e.g., Spring: March-May).

### Statistical analyses

We generated descriptive statistics for CDC-reported Lyme disease and Google Health Trends query fractions. We stratified statistics by region (see Table 1 for region definitions) and three-year time periods to show potential spatiotemporal variation. Although CDC had not yet published 2020–2021 Lyme disease data, we summarized the Google Health Trends data over this period to illustrate search trends during the COVID-19 pandemic. We also calculated each DMA’s percent change in query fractions between 2011 and 2019 to estimate where search interest has been changing. We excluded 2020–2021 from this calculation to remove the potential impact of the pandemic. Due to some interannual fluctuation in query fractions, we estimated the percent change between 2011 and 2019 using 2011 as the start value and a linear weighted moving average of 2017–2019 as the end value. In other words, 2019 was weighted the most and 2017 was weighted the least, in a linear fashion. This was done to produce a smoother–but modestly so–estimate of change in any areas where query fractions fluctuated up or down in 2019. The calculation was performed with the movavg function in the R package *pracma* [46]. To explore the hypothesis of a relationship between reported incidence rates and searches, we created choropleth maps depicting spatial variation in the individual variables for each year as well as bivariate choropleth maps showing spatial variation in the combination of reported incidence rates and searches. For the bivariate maps, natural break classification was used and adjusted slightly to ensure consistency between the three time periods.

**Table 1:** Designated market area-level characteristics between 2011 and 2021.

Characteristics	2011–2019	2011–2013	2014–2016	2017–2019	2020	2021
**Reported Lyme disease cases, n (%)**						
New England	65,658 (21.1)	24,597 (26.0)	23,551 (22.1)	17,510 (15.9)	NR	NR
Mid-Atlantic	191,556 (61.5)	52,940 (55.8)	66,528 (62.4)	72,088 (65.5)	NR	NR
Midwest	46,838 (15.0)	15,263 (16.1)	14,058 (13.2)	17,517 (15.9)	NR	NR
South	5,098 (1.6)	1,349 (1.42)	1,790 (1.7)	1,959 (1.8)	NR	NR
West	2,293 (0.7)	645 (0.7)	682 (0.6)	966 (0.9)	NR	NR
Total in United States	311,443	94,794	106,609	110,040	NR	NR
**Reported Lyme disease incidence rate (reported cases per 100,000 people), mean (SD)**						
New England	59.2 (43.9)	54.0 (33.0)	61.5 (37.5)	62.2 (58.8)	NR	NR
Mid-Atlantic	39.1 (38.9)	29.7 (31.3)	38.8 (39.6)	48.8 (42.9)	NR	NR
Midwest	9.0 (19.8)	8.7 (23.3)	8.1 (17.4)	10.2 (18.3)	NR	NR
South	0.5 (0.7)	0.4 (0.5)	0.5 (0.7)	0.5 (0.7)	NR	NR
West	0.6 (0.9)	0.6 (0.9)	0.6 (0.9)	0.7 (0.9)	NR	NR
**Google Health Trends query fraction (searches per 10 million searches), mean (SD)**						
“Lyme disease”						
New England	1,214.4 (320.4)	1,217.8 (284.8)	1,231.9 (304.8)	1,193.4 (378.7)	925.5 (150.8)	896.8 (320.1)
Mid-Atlantic	897.9 (361.2)	828.1 (346.8)	896.5 (339.1)	969.0 (386.1)	828.0 (256.7)	775.8 (301.3)
Midwest	490.9 (160.0)	440.6 (157.8)	511.8 (132.9)	520.2 (175.2)	484.8 (143.8)	436.9 (209.4)
South	368.6 (106.5)	343.7 (100.1)	402.8 (111.9)	359.4 (98.6)	351.1 (70.8)	263.2 (75.3)
West	359.9 (125.9)	324.2 (130.2)	397.9 (128.7)	357.6 (107.8)	354.9 (56.9)	266.8 (78.0)
“tick bite”						
New England	296.7 (120.5)	226.2 (57.4)	282.6 (87.6)	381.4 (145.6)	331.1 (129.8)	384.6 (188.8)
Mid-Atlantic	241.6 (112.6)	178.4 (71.3)	225.4 (82.1)	321.0 (125.7)	301.6 (98.7)	307.5 (108.4)
Midwest	148.0 (76.4)	117.8 (70.6)	136.5 (57.1)	189.6 (81.0)	174.1 (74.7)	178.8 (77.8)
South	122.2 (69.0)	104.0 (56.3)	114.6 (56.2)	148.1 (83.3)	127.9 (77.5)	118.5 (69.9)
West	70.0 (40.0)	57.5 (37.1)	68.5 (38.3)	84.1 (40.3)	75.5 (39.1)	69.0 (29.5)

New England (7 DMAs): Connecticut, Maine, Massachusetts, New Hampshire, Rhode Island, and Vermont; Mid-Atlantic (29 DMAs): Delaware, Maryland, New Jersey, New York, Pennsylvania, Virginia, West Virginia, and Washington, D.C.; Midwest (58 DMAs): Illinois, Indiana, Iowa, Kansas, Michigan, Minnesota, Missouri, Nebraska, North Dakota, Ohio, South Dakota, and Wisconsin; South (74 DMAs): Alabama, Arkansas, Florida, Georgia, Kentucky, Louisiana, Mississippi, North Carolina, Oklahoma, South Carolina, Tennessee, and Texas; West (38 DMAs): Arizona, California, Colorado, Idaho, Montana, Nevada, New Mexico, Oregon, Utah, Washington, and Wyoming. Reported Lyme disease data are from CDC. NR: Not reported at time of study.

We fit modified unadjusted and adjusted Poisson regression models to estimate the longitudinal associations between the Google Health Trends and environmental covariates and reported Lyme disease cases. Because 15% of DMAs had missing/suppressed “tick bite” query fractions due to not meeting Google’s search threshold, and “tick bite” and “Lyme disease” query fractions were strongly correlated (ρ = 0.75), we focused our regression analysis on “Lyme disease” query fractions. We dropped the four DMAs with missing “Lyme disease” query fractions, which only contributed four reported Lyme disease cases over the 2011–2019 period. Regression inference was based on a generalized estimating equation (GEE) approach [47] to account for the repeated outcomes in each DMA and any time-varying covariates (e.g., query fractions and NDVI). We used population size as the regression offset to model the rate of Lyme disease cases and an autoregressive correlation structure, which assumes that outcomes measured closer together in time are more correlated than those measured further apart. When fitting the adjusted model, we assessed all covariates for multicollinearity using correlation matrices and the variance inflation factor (VIF). If multicollinearity was indicated, we retained variables that were more strongly associated with the outcome. All significant variables from the unadjusted models were included in the adjusted model unless multicollinearity was an issue.

We assessed residual spatial autocorrelation in each year by comparing the Moran’s I statistic on the regression residuals from the population offset-only model (i.e., no covariates), the unadjusted model with “Lyme disease” query fractions, and the adjusted multivariate model. Moran’s I is a commonly used statistic to assess spatial autocorrelation for data aggregated to an areal unit, like the analysis here at the DMA geography [48]. When attempting to reduce residual spatial autocorrelation in a model, it may be appropriate to incorporate spatial lag effects, which can help capture spillover effects of adjacent geographies. In addition to covariates defined for each DMA (focal effect), we included spatial lagged covariates defined as the average of each covariate in the immediate adjacent DMAs (spatial lag effect). Functions of the x- and y-coordinates may also reduce residual spatial autocorrelation when large-scale spatial trends are present. We considered linear and spline-based forms of the DMA centroid x- and y-coordinates as covariates, to account for any general north-south and east-west trends. We further explored geographic lack of fit of the model using local Moran’s I (i.e., a local indicator of spatial association), which identifies DMAs and their neighbors that have model residuals that are significantly similar in magnitude (i.e., high-high, low-low, high-low, or low-high) [48].

Although our study area was the contiguous United States, we performed a sensitivity analysis limiting the modeled areas to DMAs in states with known high Lyme disease risk. No or low risk was approximated by the percent of state’s counties with recorded tick populations. We obtained county-level *Ixodes* tick status from CDC [49] and recoded counties as either “reported/established” or “no records.” As no uniform or truly randomized tick sampling regime exists in the United States, these statuses are based on data voluntarily collected and reported by counties and should not definitively be interpreted as tick presence or absence [49]. As DMAs comprise multiple counties that may have different statuses, for each state we calculated the percent of counties with recorded ticks and identified DMAs in states with low percentages. We reran models several times by removing DMAs in states with 0% counties reporting ticks (12 DMAs in 5 states removed), <10% (17 DMAs in 7 states removed), < 25% (26 DMAs in 11 states), and <50% (56 DMAs in 15 states removed), to check how much geography was a driving factor for modeled associations.

We performed all statistical analyses in R version 4.1.0 with the *sf*, *sfdep*, and *geepack* packages [50–53] and created all maps in ArcGIS Pro version 3.1 using DMA boundaries obtained from the Environmental Systems Research Institute (Esri) [45, 54].

## Results

### Descriptive statistics

**Fig 1:**
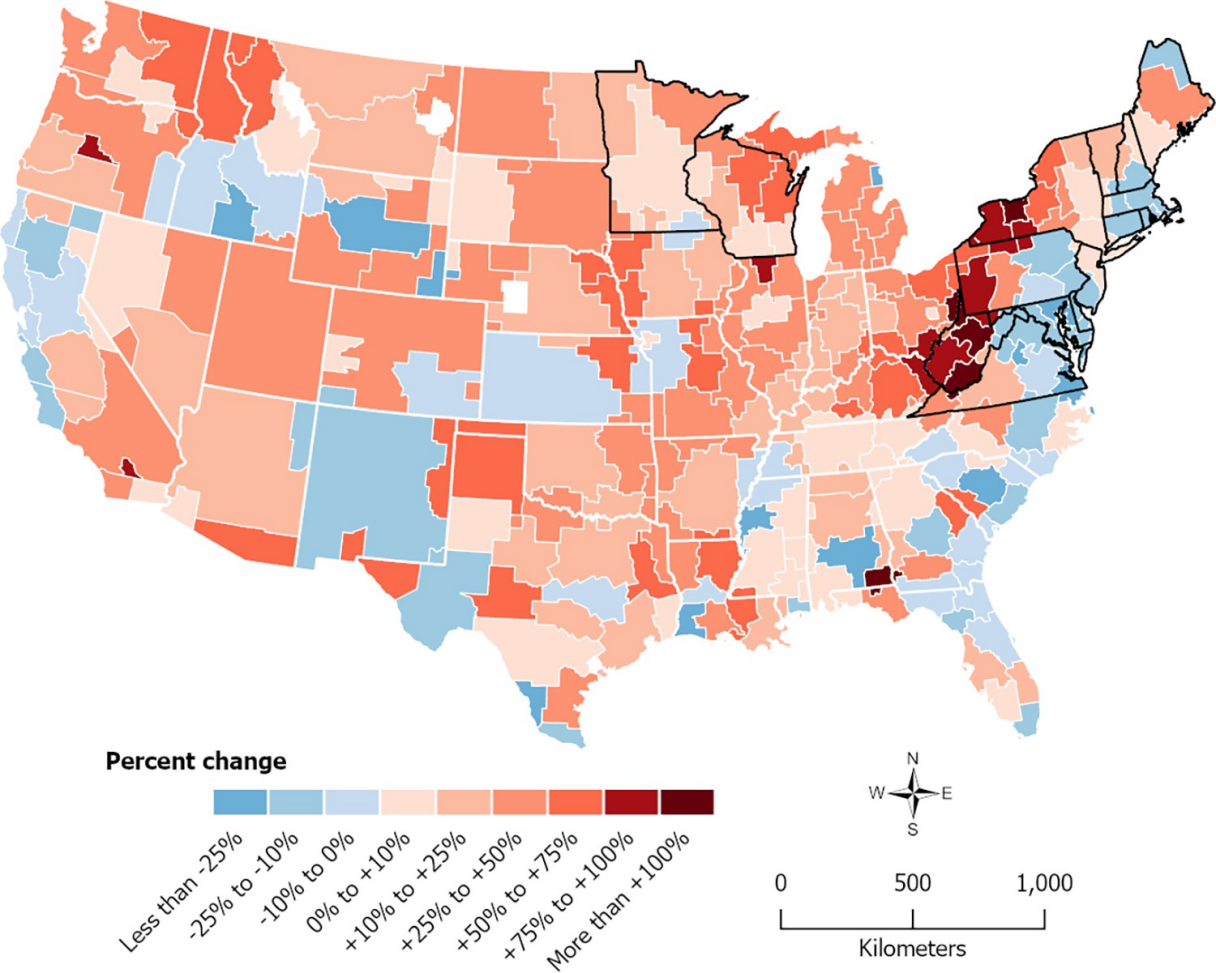
Designated market area-level percent change in Google Health Trends “Lyme disease” query fractions between 2011 and 2019. State boundaries from the Census Bureau (public domain) and high-incidence state boundaries (black) are shown for reference. DMA boundaries are the intellectual property of Esri and are used herein with permission. Copyright © 2024 Esri and its licensors. All rights reserved.

Table 1 presents summary statistics for the “Lyme disease” and “tick bite” search queries as well as reported Lyme disease cases and incidence rates. Between 2011 and 2019, searches for “Lyme disease” were most popular in the New England and Mid-Atlantic regions, averaging query fractions of 1,214.4 and 897.9, respectively. Across three-year periods, “Lyme disease” query fractions generally increased in New England, the Mid-Atlantic, and the Midwest, although Fig 1 shows marked spatial variation in the rates of change over the 2011–2019 period. States outlined in black are classified by CDC as “high-incidence” for Lyme disease [55]. Several DMAs in this high-incidence region experienced relative stability or even small decreases in searches ranging from -6% to -35% with an average of -16%. Although much of the United States posted increases in searches, a spatially contiguous grouping of DMAs showing percent increases above 50% is clearly seen in Fig 1. This darker red grouping is located on the western side of–and just beyond the border of–the northeastern region of high-incidence states outlined in black. This area represented a south-west expansion of the geographic region of relatively high “Lyme disease” query fractions over the 2011–2019 period. The top three DMAs in this area were Wheeling, West Virginia-Steubenville, Ohio (+386%), Clarksburg-Weston, West Virginia (+211%), and Bluefield-Beckley-Oak Hill, West Virginia (+114%).

Query fractions for “tick bite” were smaller overall compared to those for “Lyme disease” and the regional differences were not as pronounced. Searches consistently increased in all regions and only 16 DMAs posted decreases of more than 10% over the 2011–2019 period. In maps of DMA-level searches, the geographic area of relatively high “tick bite” searches spanned New England, the Mid-Atlantic, the Upper Midwest, and part of the South, and was not confined to the known geographic foci of Lyme disease.

Searches for “Lyme disease” generally decreased during the first year of the COVID-19 pandemic. When comparing 2020 query fractions to the 2017–2019 average; there were nearly across-the-board decreases among DMAs located in high-incidence states, with about one-third of these DMAs dipping more than 25% from their 2017–2019 average. The trend was less consistent from 2020 to 2021; searches further decreased in about half of the DMAs in high-incidence states and either remained stable or increased slightly in the other half. In the South and West, average query fractions ticked down only slightly in 2020 but showed a larger drop in 2021.

**Fig 2:**
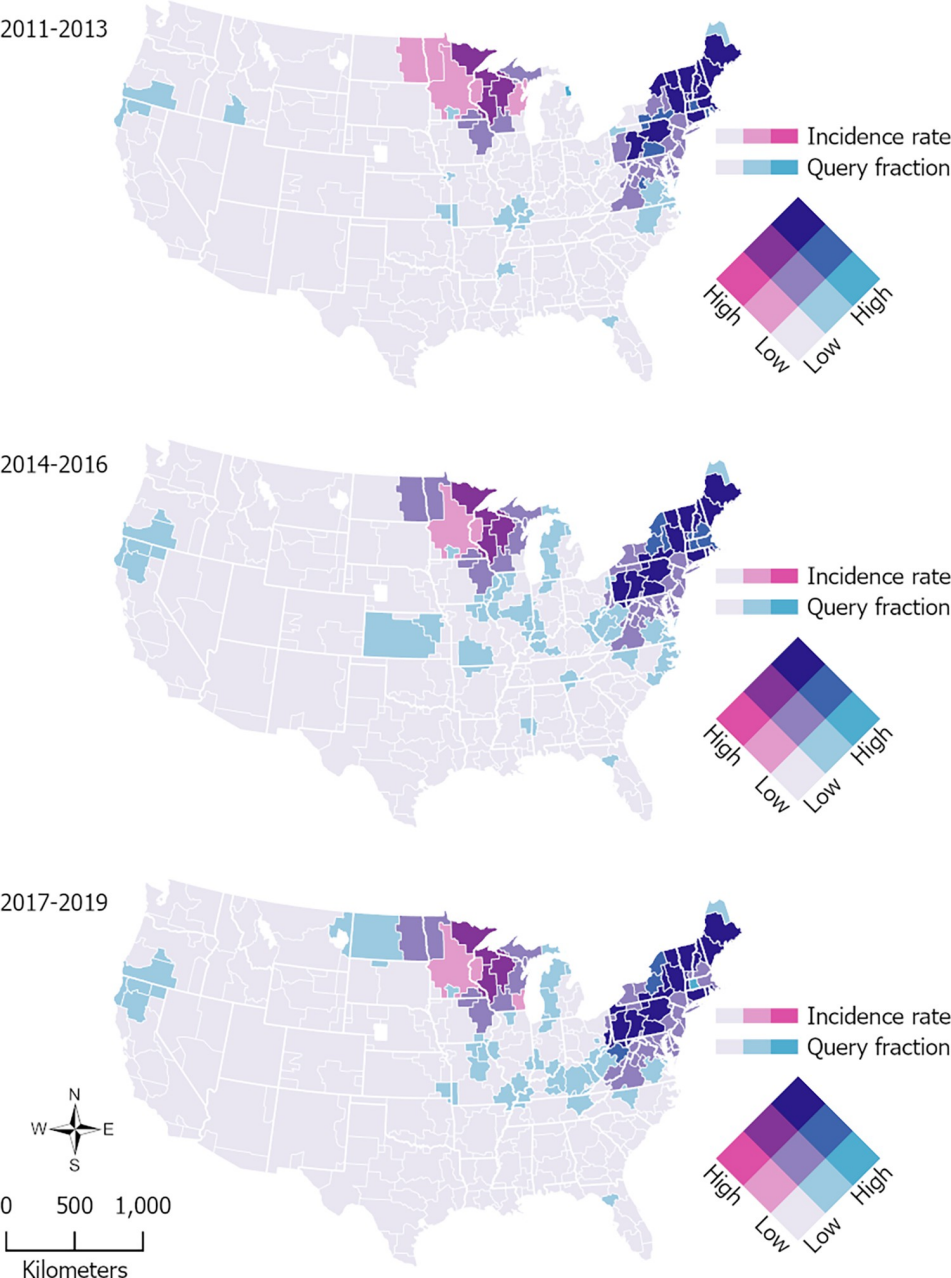
Designated market area-level spatial covariation in reported Lyme disease incidence rates and Google Health Trends “Lyme disease” query fractions using 2011–2013, 2014–2016, and 2017–2019 averages. Breaks for low, moderate, and high incidence are 0–10, 10–50, and 50+; those for query fractions are 0–500, 500–1,000, and 1,000+. State boundaries from the Census Bureau (public domain) are shown for reference. DMA boundaries are the intellectual property of Esri and are used herein with permission. Copyright © 2024 Esri and its licensors. All rights reserved.

Fig 2 explores spatial covariation in “Lyme disease” query fractions and reported incidence rates. The maps use a three-by-three classification system to classify DMAs as low, moderate, or high for each variable. The breaks for low, moderate, and higher reported incidence were 0–10, 10–50, and 50+, and those for query fractions were 0–500, 500–1,000, and 1,000+. The resulting bivariate maps show nine categories ranging from a low-low to a high-high combination of values. The three maps generally depict agreement between the two variables, but there are some notable differences. There were rare instances of high-low combinations; one of these was the Springfield-Holyoke, Massachusetts DMA in the 2017–2019 period and was the result of Massachusetts not reporting cases to CDC, thereby producing a high query fraction-low reported incidence rate combination. In all three time periods, there were DMAs in Minnesota and Wisconsin with either low query fraction-moderate reported incidence rate combinations or moderate query fraction-high reported incidence rate combinations, suggesting that searches were lower than expected given the number of cases reported.

Of special interest in Fig 2 were any DMAs with moderate query fraction-low reported incidence rate combinations, i.e., areas where Google Health Trends produced a signal that was stronger than expected and may indicate a leading edge. This is where the progression across the three time periods becomes obvious with the appearance–and spread–of spatially contiguous moderate-low DMAs. Interestingly, while searches were lower than expected in parts of Minnesota and Wisconsin, they were higher than expected on the western side of Michigan in the 2014–2016 and 2017–2019 time periods. DMAs in Virginia, West Virginia, Kentucky, Ohio, and North Carolina also showed moderate query fraction-low reported incidence rate combinations in the 2014–2016 and 2017–2019 time periods. Lastly, a grouping of moderate-low DMAs in southern Oregon and northern California remained consistent in all three time periods.

### Statistical analyses

Table 2 presents results of the unadjusted and adjusted regression analyses. For reference, an extended summary of all model covariates is provided in S2 Table as a supplement to the Google Health Trends-focused descriptive statistics in Table 1. Given that query fractions ranged up to 2,021.6 searches per 10 million searches, the relative risk (RR) is given per a 100-unit increase for a more practical interpretation. In an unadjusted analysis, “Lyme disease” query fractions were significantly associated with reported Lyme disease incidence rates (RR = 1.16, 95% CI: 1.12, 1.19); a 100-unit increase in the search rate was associated with a 16% increase in Lyme disease incidence rates. Incidence rates were also significantly associated with several environmental variables; a 3% decline in incidence rates per 25-meter increase in elevation (RR = 0.97, 95% CI: 0.95, 0.99), a 5% increase per 1% increase in percent deciduous forest cover (RR = 1.05, 95% CI: 1.03, 1.06), an 11% increase per 1% increase in percent mixed forest cover (RR = 1.11, 95% CI: 1.08, 1.14), a 17% increase per 1% increase in percent open space developed cover (RR = 1.17, 95% CI: 1.09, 1.26), an 8% increase per 0.01 increase in annual maximum NDVI (RR = 1.08, 95% CI: 1.06, 1.10), a 1% increase per one-inch increase in summer precipitation (RR = 1.01, 95% CI: 1.01, 1.02), and a 3% decline per 1°F increase in spring temperature (RR = 0.97, 95% CI: 0.96, 0.98) in unadjusted analyses. The spatial lag effect of “Lyme disease” query fractions (RR = 1.16, 95% CI: 1.11, 1.21) was significant and similar to the focal effect. S3 Table summarizes the unadjusted results for all spatial lag covariates. When fitting a multivariate model, multicollinearity among environmental variables prohibited inclusion of all significant covariates. For example, elevation and NDVI were strongly negatively correlated (ρ = -0.69), and seasonal temperature variables were all strongly positively correlated (0.75 ≤ ρ ≥ 0.90). “Lyme disease” query fractions remained significant (RR = 1.10, 95% CI: 1.07, 1.12) after adjusting for several environmental covariates. A 100-unit increase in the search rate was associated with a 10% increase in Lyme disease incidence rates in the adjusted model. The association between incidence rates and deciduous forest (RR = 1.01, 95% CI: 1.00, 1.03), open space developed cover (RR = 1.08, 95% CI: 1.01, 1.15) and maximum NDVI (RR = 1.06, 95% CI: 1.04, 1.08) also remained significant. Mixed forest and precipitation variables were no longer significant in the adjusted model, but the direction and size of the effects were comparable to the unadjusted models, with mixed forest showing a larger drop in effect size. VIF did not exceed 7 in the adjusted model, indicating no serious multicollinearity.

**Table 2:** Unadjusted and adjusted relative risks (RR) and 95% confidence intervals (CI) for designated market area-level reported Lyme disease incidence rates (n = 202).

	Unadjusted			Adjusted^d^		
Characteristics	RR	95% CI	*P* value	RR	95% CI	*P* value
**Focal effects**						
“Lyme disease” Google Health Trends query fraction (searches/10 million searches)^a^	1.16	(1.12, 1.19)	<0.001	1.10	(1.07, 1.12)	<0.001
Elevation (m)^b^	0.97	(0.95, 0.99)	0.004			
Deciduous forest cover (%)	1.05	(1.03, 1.06)	<0.001	1.01	(1.00, 1.03)	0.047
Mixed forest cover (%)	1.11	(1.08, 1.14)	<0.001	1.02	(0.99, 1.05)	0.173
Open space developed (%)	1.17	(1.09, 1.26)	<0.001	1.08	(1.01, 1.15)	0.024
Maximum NDVI (multiplied by 100)	1.08	(1.06, 1.10)	<0.001	1.06	(1.04, 1.08)	<0.001
Precipitation (in)						
Winter	1	(1.00, 1.00)	0.520			
Spring	1.01	(1.00, 1.01)	0.007	1.00	(1.00, 1.01)	0.170
Summer	1.01	(1.01, 1.02)	0.001	1.01	(1.00, 1.02)	0.167
Fall	0.99	(0.99, 1.00)	0.037	0.99	(0.99, 1.00)	0.065
Annual	1	(1.00, 1.00)	0.520			
Average temperature (°F)						
Winter	0.99	(0.98, 1.00)	0.092			
Spring	0.97	(0.96, 0.98)	<0.001			
Summer	0.94	(0.91, 0.96)	<0.001			
Fall	1.01	(0.99, 1.04)	0.170			
Annual	0.96	(0.94, 0.98)	<0.001			
Centroid x-coordinate (km)^b^	1.04	(1.02, 1.05)	<0.001			
Centroid y-coordinate (km)^b^	1.05	(1.03, 1.07)	<0.001			
**Spatial lag effects**						
“Lyme disease” Google Health Trends query fraction (searches/10 million searches)^a^	1.16	(1.11, 1.21)	<0.001			
“tick bite” Google Health Trends query fraction (searches/10 million searches)^b^^,^^c^	1.07	(1.04, 1.10)	<0.001			

^a^Relative risk is given per 100-unit increase.

^b^Relative risk is given per 25-unit increase.

^c^Not included in focal model due to missing focal observations.

^d^Adjusted model includes a cubic spline of the centroid y-coordinate with four degrees of freedom.

The Moran’s I statistic for the offset-only model was significantly positive for each year and ranged between 0.60 and 0.78, indicating that adjacent DMAs had more similar Lyme disease incidence rates compared to non-adjacent DMAs. The Moran’s I statistic for the unadjusted model decreased but remained significantly positive for each year (0.55–0.67), indicating spatial autocorrelation in the residuals after accounting for “Lyme disease” search rates. The Moran’s I statistic for the adjusted model decreased further (0.44–0.52) but remained significantly positive. Further attempts were made to reduce residual spatial autocorrelation by including spatial lagged covariates, but multicollinearity between focal and lagged variables was a problem. We also experimented with functions of the DMA centroid x- and y-coordinates, keeping in mind that some environmental covariates already captured north-south or east-west trends. For example, there was a very strong north-south trend in spring temperature, with warmer temperatures in the south and cooler temperatures in the north. NDVI and summer precipitation especially showed strong east-west trends, with more vegetation density and precipitation in the east. NDVI was strongly correlated with the x-coordinate (ρ = 0.71) and had a stronger association with the outcome. After comparing how different models reduced the Moran’s I statistic, we ultimately included a natural cubic spline of the centroid y-coordinate with four degrees of freedom and excluded spring temperature due to multicollinearity (ρ = 0.94). The Moran’s I statistic ranged from 0.38 to 0.48, which was smaller compared to the model without the y-coordinate centroid. Although residual spatial autocorrelation persisted in the adjusted model, indicating possible model misspecification, the modified Poisson (GEE) regression inference was based on a quasi-Poisson approach with robust standard errors accounting for overdispersion and was likely sufficient to provide proper inference with the remaining residual spatial autocorrelation.

When analyzing the model residuals via local Moran’s I, we identified significant local clusters of high residuals, or regions where the model underestimated the outcome in a particular year. S2 Fig shows an example local Moran’s I cluster map for 2019; red areas indicate areas with high residuals surrounded by other areas with high residuals. The location and extent of the clusters varied from year to year but frequently appeared in almost all of Pennsylvania and Maryland and parts of Wisconsin and Minnesota (Minnesota not shown in 2019).

The sensitivity analysis restricted to areas with recorded tick presence (S4 Table) had a small impact on the modeled relative risks and significance levels of some covariates, but the association between “Lyme disease” search rates and incidence rates (RR = 1.09, CI: 1.07, 1.12) remained highly significant. The removed DMAs contributed few reported Lyme disease cases, which was expected given their tick status. Even with a 50% restriction (i.e., included DMAs must be in states with at least 50% counties reporting ticks), the removed DMAs only contributed about 1% of total cases over the study period. The large number of cases reported in endemic areas appeared to drive the associations between Lyme disease, Google searches, and environmental factors.

## Discussion

### Google Health Trends reveals a geographic leading edge

We hypothesized that the geographic leading edge of Lyme disease in the United States may be discernable from Google Health Trends data. Mapping changes in “Lyme disease” search interest between 2011 and 2019 (Fig 1) revealed an expanding area of higher interest occurring along the edges of the northeastern focus of the disease, supporting this hypothesis. The south-west expansion of Lyme disease in and from this region over time is evident from larger increases in searches in western New York, western Pennsylvania, West Virginia, and even Ohio and Kentucky. In areas of Michigan, Ohio, Virginia, West Virginia, Kentucky, and North Carolina, Google Health Trends data also produced a signal in recent years that was stronger than expected when compared to CDC-reported incidence (Fig 2). This may indicate that internet search patterns can pick up the leading edge before passive surveillance. Taken together, Figs 1 and 2 suggest that risk may be increasing along the edges of the Northeast and Upper Midwest foci of Lyme disease.

Several of the abovementioned states are noteworthy as they are not considered high-risk states based on reported case data. Despite historically low reported incidence compared to neighboring Wisconsin, the spread of Lyme disease into Michigan’s Upper Peninsula and along Lake Michigan is documented [56], and search data suggest that incidence in the state could be underreported. North Carolina is also significant because it is thought to be on the leading edge of the southern expansion of Lyme disease [57, 58]. Although the expansion of Lyme disease into Virginia is documented by surveillance data, Lantos et al. [57] point out that North Carolina is still considered a low-incidence state based on reported case numbers. However, they predict endemic transmission in the state in coming years based on current trends. Similarly, companion animal disease data collected by the Companion Animal Parasite Council [59] forecast that Lyme disease risk is increasing in North Carolina. Both Figs 1 and 2 show that Google data agree with these projections, with the Greensboro-High Point-Winston Salem DMA bordering southwestern Virginia standing out. Higher than expected searches in the state could suggest that not all cases were picked up by CDC surveillance in recent years, perhaps due to low clinician awareness leading to underdiagnosis and underreporting. We should also note Ohio, which has seen increasing Lyme disease cases in eastern counties. Its leading edge status appears less remarked on in the literature, although an increase in *Ixodes scapularis* ticks in eastern Ohio has been documented and attributed to the east-west expansion of ticks from Pennsylvania [60]. In the Google data, it is difficult to isolate eastern Ohio as the DMAs in that area overlap part of West Virginia and Pennsylvania. However, together with Fig 1, which showed a large growth in search rates in that area, the Google data indicate that eastern Ohio is an area to watch.

The more recent trends in leading edge states should be contrasted with southern Oregon and northern California, which in Fig 2 consistently showed a stronger than expected signal. Although these states are considered low-incidence by CDC, cases tend to be reported in counties in southern Oregon and northern California [35]. That this region always showed low reported incidence rates but moderate query fractions could indicate that cases were underreported between 2011 and 2019. This is less suggestive of a leading edge appearing over time; instead, Google data may be picking up an area with stable Lyme disease but consistent underreporting.

### Predictive power of “Lyme disease” searches and model limitations

Multivariate regression analysis supported a statistically significant longitudinal association between “Lyme disease” search rates and CDC-reported incidence rates after controlling for environmental factors (Table 2). Together with the exploratory findings on the geographic expansion of searches, this demonstrates the potential value of Google Health Trends as a source of epidemiological data in the study of Lyme disease.

The findings of the regression analysis should be interpreted with care given the ecologic nature of the study and the size of the spatial unit of analysis. The modifiable areal unit problem (MAUP) arises when spatial data are aggregated to areal units and the results of statistical analyses are contingent on the configuration of those units [61]. Because the smallest geography for Google Health Trends data is the DMA, we had to aggregate county-level Lyme disease data to this larger geography. Traditionally used in the media and advertising industries, DMAs are groupings of counties that combine metropolitan and non-metropolitan areas and are not necessarily the most appropriate geography for studying tickborne diseases, which do not follow administrative boundaries.

Despite finding significant associations, the regression analysis revealed a geographic lack of fit in some high-incidence Lyme disease regions, as shown by the persistence of local spatial clustering (S2 Fig) after accounting for Google searches and environmental risk factors. There could be several reasons why the model did not perform well in certain areas. Perhaps the Google Health Trends data did not produce strong enough of a signal there. For example, we mentioned that search rates in Minnesota and Wisconsin tended to be more moderate compared to those in other high-incidence states, which can be seen in Fig 2. The model’s underestimation of Lyme disease incidence in these states in certain years was unsurprising. The clusters in Pennsylvania and Maryland were less expected. Although Fig 2 showed agreement between search rates and reported incidence rates in these states, there is a certain degree of oversimplification in bivariate maps. For example, Pennsylvania did have high search rates but other areas with similar incidence rates had even higher search rates. There could also be other variables and spatial processes not captured by the model that led to clusters of unexplained variability. The analysis was further limited by the use of passive Lyme disease surveillance data for the modeled outcome. CDC case reporting is based on county of residence, not county of exposure. More importantly, cases are known to be underreported, and reporting practices may vary by county and state, and from year to year. If the outcome was not uniformly and accurately measured across the study area due to reporting differences and inevitable underreporting, this could contribute to the model’s geographic lack of fit. In addition to computing overall estimates of the associations between the outcome and each covariate, we could have explored effect modification by region. However, there were only 202 DMAs with Google data, so we did not pursue a stratified analysis due to sample size considerations. Future work could also explore more localized relationships between the outcome and covariates using alternative modeling approaches, such as geographically weighted regression, generalized additive models, and mixed effect models [62, 63].

The purpose of our modeling efforts was to determine if the association between Google searches and CDC-reported incidence rates was significant and remained significant when controlling for landscape and climate differences across DMAs. Although our objective was not to advance understanding of environmental patterns on disease risk, which has been the focus of other research conducted at more appropriate spatial scales, our results were generally consistent with the literature [17, 37–41]. Elevation was negatively associated with Lyme disease incidence rates and NDVI, percent forest cover, and percent open space vegetation were positively associated. As Moon et al. [38] note, non-forest vegetation on its own may not carry a high entomologic risk but may represent edge habitats between forest and residential lawn grasses, parks, and recreation areas that may favor tick encounters. Further exploration of this hypothesis is better suited for smaller scale studies. Temperature was negatively associated with incidence rates, which is consistent with other research in which the study area is the eastern or contiguous United States [39] and temperatures vary considerably by region.

### Geographic specificity of search queries

That higher search rates for “tick bite” were more geographically spread out was an interesting, albeit logical, finding. Tick bites are not specific to Lyme disease and may relate to other non-*Ixodes* species of ticks that have different geographic distributions. Even among the vectors for Lyme disease, *Ixodes scapularis* and *Ixodes pacificus*, the tick habitat is known to be more geographically widespread than infection [64, 65]. Exploring both “Lyme disease” and “tick bite” searches helped demonstrate the geographic specificity of search queries and allowed us to select the more focal query for our analysis. This may be a consideration for other researchers using the Google Health Trends API in spatial epidemiology applications.

### Impact of the COVID-19 pandemic

We found that Lyme disease-related searches decreased in 2020, which is consistent with limited data that have been published about the impact of the pandemic on disease risk and healthcare-seeking behavior. According to McCormick et al. [5], although survey data suggest that Americans spent more time outdoors during the pandemic, both emergency department visits for tick bites and laboratory testing for Lyme disease decreased. It is unlikely that this reflected a true decrease in the risk of acquiring Lyme disease and more likely that the pandemic altered concern and healthcare-seeking behavior–both motivators for Google searching–for non-COVID-19 issues. There is also some evidence of misdiagnosis of Lyme disease as COVID-19 early in the pandemic due to overlapping non-specific symptoms [66, 67]. It is conceivable that, at a time when the country was on high alert for COVID-19, people experiencing flu-like symptoms would not have thought to search for Lyme disease.

### Limitations of Google Health Trends

A limitation of Google Health Trends as a data source is the risk of overinterpreting the clinical significance of searches. We never know the context behind a user searching for Lyme disease-related queries; it is impossible to distinguish between general curiosity or concern and diagnosis-seeking. Although higher “Lyme disease” query fractions were, as expected, concentrated in the Northeast, Mid-Atlantic, and Upper Midwest between 2011 and 2019, Fig 1 showed that search interest grew across much of the country during this period, which could reflect increased Lyme disease risk as well as simply increased awareness and curiosity about the disease. Although Google Health Trends data largely showed what we hypothesized, some results were a challenge to interpret. For example, it is unclear why the signals produced in Minnesota and Wisconsin were more moderate compared to other high-incidence states. It is also difficult to interpret the modest decreases in search interest that a few traditionally high-incidence DMAs experienced over time. We suspect in both cases that the different demographic makeups of DMAs, including age, urbanicity/rurality, internet access and usage, and health information-seeking behaviors, could have played a role. For example, perhaps in areas of long-established risk, residents were already more aware of Lyme disease and turned less frequently to Google, and the Google Health Trends results should not be interpreted as signaling lower or reduced risk.

It is worth restating here the limitations of DMAs as a spatial unit of analysis in public health applications. DMAs may over-aggregate differences over the urban-rural continuum, including underlying Lyme disease incidence patterns. Making Google Health Trends data available at county or even ZIP code levels would not only allow for improved comparisons with public health data sets but also increase specificity when identifying emerging risk areas for disease. In fact, researchers at Google previously predicted county-level Lyme disease risk using licensed search data with more detailed information about individual user sessions [68]. Although the project does not appear to have continued, it shows that Google Health Trends may not capture the full capabilities and richness of Google’s data. Of course, spatial granularity must be weighed against the privacy of search engine users. As seen in the “Lyme disease” and “tick bite” searches, Google’s privacy threshold already produces some missingness at the DMA level in relatively low-incidence geographic areas where there are not enough searches. This missingness would likely increase if data were made available at the county level, but at the same time, more granular data could enhance disease surveillance where it counts most, which is emerging counties with increasing searches.

### Enhancing traditional disease surveillance

The burden of Lyme disease in the United States is increasing, but insufficient monitoring and surveillance systems hinder the identification of emerging risk areas and contribute to a lack of awareness among the public and health care providers. No single surveillance mechanism is perfect, so effective disease tracking should integrate multiple data streams and balance the strengths and limitations of each. Google Health Trends data are consistent with prior publications and reports relying on insurance claims, electronic health records, and human and veterinary laboratory testing data [2, 59, 69, 70] and should be considered as a complementary tool in Lyme disease surveillance. Internet-based data present opportunities for accessibility and timeliness. Although the data used in this study are not publicly accessible, researchers can easily obtain Google Heath Trends data with an API key from Google, and comparable, though coarser, data are available from the public Google Trends website. All Google Trends data can be tracked in near real time, whereas the publication of passive surveillance data can be delayed by multiple years. Attention should be given to where Google Health Trends data are producing interesting signals. For example, this study shows a large growth in search interest in parts of Ohio and West Virginia since 2011 as well as more recent changes in Michigan and North Carolina. These findings may point to areas where public and physician awareness of Lyme disease should be increased. As Boyce et al. [58] note, clinicians in emerging areas like North Carolina may be unfamiliar with Lyme disease, leading to delayed diagnosis and treatment. Our findings may also point to areas where other surveillance efforts should be strengthened to confirm actual changes in acarological risk. In southern Oregon and northern California, they may indicate a need to address suspected underreporting and lack of diagnostic recognition of Lyme disease by physicians in areas labeled as low-incidence.

As a final note, CDC implemented a revised Lyme disease case definition in 2022 that applies to high-incidence states only. The new criteria make it less burdensome for these states to report cases based on positive laboratory testing alone, without the need for clinical follow-up. Low-incidence states are still required to produce positive laboratory evidence and clinical information. Preliminary data under the new requirements are beginning to be published and indicate a sharp increase in cases reported from high-incidence jurisdictions and more mixed results from low-incidence ones. However, it is difficult to compare these data to prior years and make any assumptions about changes in disease risk [71]. Moving forward, it is unclear how the new surveillance criteria will affect the ability to detect the leading edge of geographic expansion. Cases may be undercounted in low-incidence states, even in areas with higher or increasing incidence. If anything, the changes in surveillance strengthen the argument that multiple data sources should inform our understanding of where Lyme disease is spreading.

## Conclusions

This study complements prior works that have found Google search data to be a reliable source of epidemiological data in the study of Lyme disease. It is novel is two respects: first, we used the Google Health Trends API to extract raw annual search rates at a small geographic scale (DMAs), building on an existing literature that largely has used the coarser, indexed data available from the public version of Google Trends–which is not appropriate for spatiotemporal analysis–and focused on country-level temporal trends without considering sub-country geographic variation. Second, it is the first study to investigate spatial trends in Lyme disease-related searches over time with the aim of identifying emerging risk areas.

Our findings demonstrate the potential of Google Health Trends to track spatiotemporal patterns in Lyme disease and help identify the geographic leading edge of disease risk. Although Google Health Trends cannot replace traditional surveillance methods, it should be integrated with other data sources to identify geographic areas that are becoming endemic for Lyme disease. The public health contribution of Google Health Trends data may be significant in informing the public and health care providers about emerging risks in their geographic regions.

## Supporting information

S1 Table Proportional assignment of county-level Lyme disease cases to designated market areas in split counties. (PDF)

S2 Table Extended descriptive statistics for model covariates. (PDF)

S3 Table Unadjusted relative risks (RR) and 95% confidence intervals (CI) for designated market area-level reported Lyme disease incidence rates with spatial lag effects (n = 202). (PDF)

S4 Table Adjusted relative risks (RR) and 95% confidence intervals (CI) for designated market area (DMA)-level reported Lyme disease incidence rates: Sensitivity analysis restricted to DMAs in states with at least 50% counties reporting ticks (n = 146). (PDF)

S1 Fig Distribution of coefficient of variation across different Google Health Trends sampling size groups. (TIF)

S2 Fig Local Moran’s I cluster map of the adjusted model residuals for 2019. State boundaries from the Census Bureau (public domain) are shown for reference. DMA boundaries are the intellectual property of Esri and are used herein with permission. Copyright © 2024 Esri and its licensors. All rights reserved.
(TIF)
