# Supplementary material for: Identifying the geographic leading edge of Lyme disease in the United States with internet searches: A spatiotemporal analysis of Google Health Trends data
Source: PLoS One. 2024 Nov 13;19(11):e0312277. doi: 10.1371/journal.pone.0312277 (PMC11560046; doi:10.1371/journal.pone.0312277)
Supplement: S1 Table — (PDF) [file pone.0312277.s001.pdf]

S1 Table. Proportional assignment of county-level Lyme disease cases to designated market areas in split counties.

County	State	DMA	Proportion of County in DMA (%)	Number of Cases Assigned to DMA (2011-2019)
Apache	AZ	Phoenix, AZ	29	0
Apache	AZ	Albuquerque-Santa Fe, NM	71	1
El Dorado	CA	Reno, NV	14	0
El Dorado	CA	Sacramento-Stockton-Modesto, CA	86	11
Kern	CA	Los Angeles, CA	29	0
Kern	CA	Bakersfield, CA	71	3
Riverside	CA	Los Angeles, CA	82	29
Riverside	CA	Palm Springs, CA	18	0
Solano	CA	San Francisco-Oakland-San Jose, CA	11	0
Solano	CA	Sacramento-Stockton-Modesto, CA	89	10
Lea	NM	Albuquerque-Santa Fe, NM	69	1
Lea	NM	Odessa-Midland, TX	31	0
Oneida	NY	Utica, NY	32	101
Oneida	NY	Syracuse, NY	68	225
