# Supplementary material for: Identifying the geographic leading edge of Lyme disease in the United States with internet searches: A spatiotemporal analysis of Google Health Trends data
Source: PLoS One. 2024 Nov 13;19(11):e0312277. doi: 10.1371/journal.pone.0312277 (PMC11560046; doi:10.1371/journal.pone.0312277)
Supplement: S2 Table — (PDF) [file pone.0312277.s002.pdf]

S2 Table. Extended descriptive statistics for model covariates.

	Mean ± SD (Range)				
**Characteristic**	**New England**	**Mid-Atlantic**	**Midwest**	**South**	**West**
"Lyme disease" Google Health Trends query fraction (searches/10 million searches)	1,214.4 ± 320.4 (576.5 - 2,021.6)	897.9 ± 361.2 (221.8 - 1,955.5)	490.9 ± 160.0 (192.6 - 1,678.6)	368.6 ± 106.5 (117.5 - 747.3)	359.9 ± 125.9 (128.2 - 1,109.7)
Elevation (m)	197.0 ± 72.9 (60.5 - 292.3)	336.7 ± 186.0 (6.6 - 703.2)	350.3 ± 235.2 (134.4 - 1,589.0)	196.3 ± 259.0 (0.8 - 1,317.0)	1,177.5 ± 538.4 (193.6 - 2,105.0)
Deciduous forest cover (%)	23.1 ± 10.2 (12.7 - 44.6)	39.5 ± 17.6 (1.8 - 73.1)	16.1 ± 13.3 (0.0 - 57.0)	10.8 ± 14.0 (0.0 - 47.3)	1.1 ± 2.9 (0.0 - 17.5)
Mixed forest cover (%)	25.1 ± 7.5 (13.0 - 32.0)	11.8 ± 5.5 (3.6 - 21.7)	3.3 ± 4.3 (0.0 - 20.6)	5.7 ± 5.5 (0.0 - 20.4)	1.5 ± 2.7 (0.0 - 10.6)
Open space developed (%)	5.2 ± 2.6 (1.7 - 8.8)	5.8 ± 2.6 (3.0 - 13.2)	4.3 ± 1.9 (0.7 - 9.8)	4.5 ± 2.0 (0.8 - 10.7)	1.9 ± 1.4 (0.4 - 6.3)
Maximum NDVI	0.9 ± 0.0 (0.8 - 0.9)	0.9 ± 0.0 (0.8 - 0.9)	0.9 ± 0.1 (0.4 - 0.9)	0.8 ± 0.1 (0.2 - 0.9)	0.6 ± 0.2 (0.2 - 0.9)
Precipitation, winter (in)	11.7 ± 1.6 (8.1 - 14.9)	10.2 ± 2.4 (4.7 - 19.9)	5.9 ± 3.1 (0.8 - 18.9)	11.3 ± 6.3 (0.1 - 29.4)	8.7 ± 9.3 (0.2 - 58.4)
Precipitation, spring (in)	11.4 ± 2.8 (5.5 - 17.6)	12.2 ± 3.3 (6.1 - 22.1)	11.2 ± 3.9 (1.3 - 28.4)	13.0 ± 5.8 (0.0 - 31.7)	6.7 ± 5.8 (0.0 - 37.1)
Precipitation, summer (in)	13.5 ± 3.2 (7.2 - 21.8)	14.3 ± 3 (6.6 - 23.4)	12.5 ± 3.8 (3.6 - 24.2)	15.2 ± 7.4 (1.2 - 51.8)	2.7 ± 2.3 (0.0 - 10.1)
Precipitation, fall (in)	12.7 ± 3.6 (8.0 - 24.6)	11.7 ± 3.8 (5.3 - 23.7)	9.1 ± 3.4 (0.5 - 18.9)	11.1 ± 5.1 (1.4 - 31.0)	5.3 ± 5.2 (0.2 - 37.8)
Precipitation, annual (in)	49.2 ± 7.5 (37.1 - 69.1)	48.5 ± 8.2 (32.3 - 76.8)	38.8 ± 10.2 (8.3 - 68.6)	50.8 ± 16.8 (4.3 - 92.9)	23.2 ± 18.3 (3.1 - 113.1)
Average temperature, winter (°F)	24.1 ± 6.8 (10.4 - 36.4)	30.7 ± 6.9 (14.1 - 46.2)	25.7 ± 7.4 (0.4 - 40.8)	48.8 ± 7.8 (30.7 - 71.4)	35.5 ± 11.2 (13.4 - 58.7)
Average temperature, spring (°F)	43.2 ± 4.5 (33.7 - 52.1)	49.4 ± 5.6 (37.5 - 61.7)	48.4 ± 6.0 (32.3 - 64.1)	64.7 ± 5.3 (52.0 - 78.4)	49.4 ± 8.5 (33.7 - 72.8)

Average temperature, summer (°F)	67.3 ± 2.9 (62.5 - 71.8)	70.5 ± 3.7 (63.7 - 79.0)	71.5 ± 3.7 (61.4 - 82.4)	80.6 ± 3.0 (72.7 - 90.5)	69.3 ± 7.3 (57.4 - 92.7)
Average temperature, fall (°F)	49.6 ± 3.9 (40.7 - 56.5)	53.5 ± 4.3 (44.6 - 64.3)	51.8 ± 4.7 (38.2 - 63.2)	66.1 ± 5.3 (54.3 - 80.5)	53.3 ± 9.1 (36.5 - 76.4)
Average temperature, annual (°F)	46.1 ± 4.2 (38.1 - 53.1)	51.1 ± 4.8 (41.8 - 61.5)	49.4 ± 5.0 (35.3 - 60.4)	65.1 ± 4.9 (53.5 - 77.9)	51.9 ± 8.6 (36.8 - 74.6)
