# Supplementary material for: Identifying the geographic leading edge of Lyme disease in the United States with internet searches: A spatiotemporal analysis of Google Health Trends data
Source: PLoS One. 2024 Nov 13;19(11):e0312277. doi: 10.1371/journal.pone.0312277 (PMC11560046; doi:10.1371/journal.pone.0312277)
Supplement: S3 Table — (PDF) [file pone.0312277.s003.pdf]

S3 Table. Unadjusted relative risks (RR) and 95% confidence intervals (CI) for designated market area-level reported Lyme disease incidence rates with spatial lag effects (n = 202).

	**Unadjusted**		
**Characteristics**	**RR**	**95% CI**	***P* value**
**Spatial lag effects**			
"Lyme disease" Google Health Trends query fraction (searches/10 million searches)[a]	1.16	(1.11, 1.21)	<0.001
"tick bite" Google Health Trends query fraction (searches/10 million searches)[b]	1.07	(1.04, 1.10)	<0.001
Elevation (m)[b]	0.97	(0.95, 0.98)	<0.001
Deciduous forest cover (%)	1.06	(1.05, 1.07)	<0.001
Mixed forest cover (%)	1.16	(1.12, 1.20)	<0.001
Open space developed (%)	1.49	(1.29, 1.71)	<0.001
Maximum NDVI (multiplied by 100)	1.09	(1.07, 1.11)	<0.001
Precipitation (in)			
Winter	1.00	(1.00, 1.01)	0.610
Spring	1.01	(1.00, 1.02)	0.004
Summer	1.01	(1.00, 1.03)	0.031
Fall	0.99	(0.99, 1.00)	0.054
Annual	1.00	(1.00, 1.01)	0.490
Average temperature (°F)			
Winter	0.99	(0.98, 1.00)	0.049
Spring	0.97	(0.96, 0.98)	<0.001
Summer	0.93	(0.90, 0.96)	<0.001
Fall	1.01	(0.99, 1.03)	0.190
Annual	0.96	(0.94, 0.98)	<0.001

[a]Relative risk is given per 100-unit increase.
[b]Relative risk is given per 25-unit increase.
