# Supplementary material for: Identifying the geographic leading edge of Lyme disease in the United States with internet searches: A spatiotemporal analysis of Google Health Trends data
Source: PLoS One. 2024 Nov 13;19(11):e0312277. doi: 10.1371/journal.pone.0312277 (PMC11560046; doi:10.1371/journal.pone.0312277)
Supplement: S4 Table — (PDF) [file pone.0312277.s004.pdf]

S4 Table. Adjusted relative risks (RR) and 95% confidence intervals (CI) for designated market area (DMA)-level reported Lyme disease incidence rates: sensitivity analysis restricted to DMAs in states with at least 50% counties reporting ticks (n = 146).

	**Adjusted[b]**		
**Characteristics**	**RR**	**95% CI**	***P* value**
**Focal effects**			
"Lyme disease" Google Health Trends query fraction (searches/10 million searches)[a]	1.09	(1.07, 1.12)	<0.001
Deciduous forest cover (%)	1.01	(1.00, 1.03)	0.065
Mixed forest cover (%)	1.02	(0.99, 1.05)	0.236
Open space developed (%)	1.07	(1.01, 1.14)	0.033
Maximum NDVI (multiplied by 100)	1.07	(1.04, 1.09)	<0.001
Spring precipitation (in)	1.00	(1.00, 1.01)	0.152
Summer precipitation (in)	1.01	(1.00, 1.02)	0.156
Fall precipitation (in)	0.99	(0.99, 1.00)	0.059

[a]Relative risk is given per 100-unit increase.

[b]Adjusted model includes a cubic spline of the centroid y-coordinate with four degrees of freedom.
