# Supplementary figures and images for: Identifying the geographic leading edge of Lyme disease in the United States with internet searches: A spatiotemporal analysis of Google Health Trends data
Source: PLoS One. 2024 Nov 13;19(11):e0312277. doi: 10.1371/journal.pone.0312277 (PMC11560046; doi:10.1371/journal.pone.0312277)

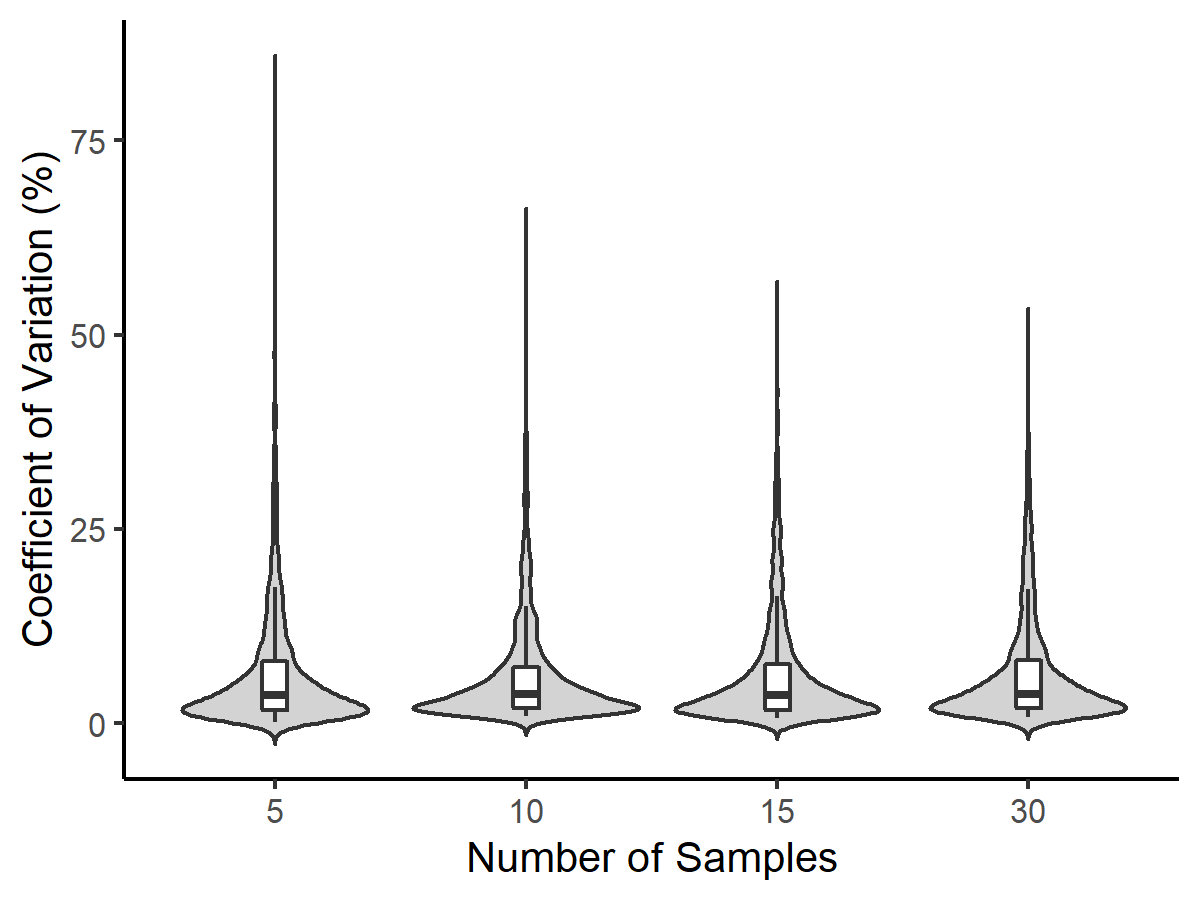

Supplement: S1 Fig — (TIF) [file pone.0312277.s005.tif]

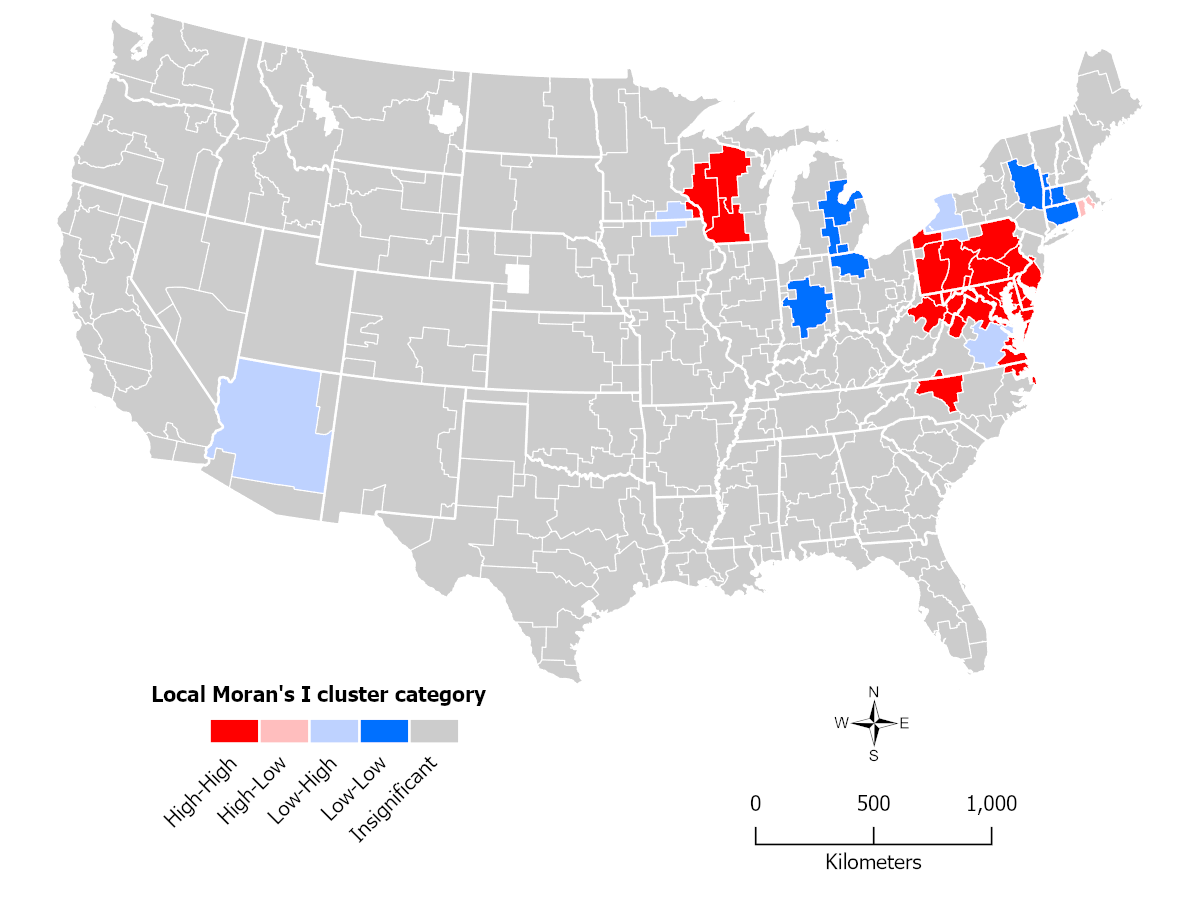

Supplement: S2 Fig — State boundaries from the Census Bureau (public domain) are shown for reference. DMA boundaries are the intellectual property of Esri and are used herein with permission. Copyright © 2024 Esri and its licensors. All rights reserved. (TIF) [file pone.0312277.s006.tif]
